# An advanced phase retrieval algorithm in N-step phase-shifting interferometry with unknown phase shifts

**DOI:** 10.1038/srep44307

**Published:** 2017-03-14

**Authors:** Jiaosheng Li, Liyun Zhong, Shengde Liu, Yunfei Zhou, Jie Xu, Jindong Tian, Xiaoxu Lu

**Affiliations:** 1 Nanophotonic Functional Materials and Devices, South China Normal University, Guangzhou 510006, China; 2 College of Optoelectronic Engineering, Shenzhen University, Shenzhen 518060, China

## Abstract

In phase-shifting interferometry with unknown phase shifts, a normalization and orthogonalization phase-shifting algorithm (NOPSA) is proposed to achieve phase retrieval. The background of interferogram is eliminated through using the orthogonality of complex sinusoidal function; and the influence of phase shifts deviation on accuracy of phase retrieval is avoided through both normalization and orthogonalization processing. Compared with the current algorithms with unknown phase shifts, the proposed algorithm reveals significantly faster computation speed, higher accuracy, better stability and non-sensitivity of phase shifts deviation.

Phase-shifting interferometry (PSI)^1^, a non-intervention, high accuracy, full field, rapid speed and quantitative phase retrieval technique, has been widely used in wavefront reconstruction, optical element testing, refractive index measurement^2^ and quantitative phase imaging (QPI) microscopy^3^,^4^,^5^,^6^, which is a tool of phase-sample evaluation in micro-scale and frequently applied in biomedicine in, e.g., red blood cell^3^ and cancer diagnosis^4^. To date, many phase-shifting algorithms (PSAs) have been developed^7^,^8^,^9^,^10^,^11^,^12^,^13^,^14^,^15^,^16^,^17^,^18^,^19^, such as in temporal PSAs, the fixed-coefficients PSAs^7^,^10^,^11^ and tunable PSAs^7^,^12^,^13^ reveal rapid speed and high accuracy, but their performance is usually affected by the phase shifts deviation (PSD) induced by the detuning of phase shifter. Besides, two asynchronous temporal PSAs are proposed, including the self-tuning PSAs^14^,^15^,^16^, in which the phase demodulation is performed from a sequence of phase-shifting interferograms with unknown but constant phase shifts; the self-calibrating PSAs^17^,^18^,^19^, in which the phase is calculated from a sequence of phase-shifting interferograms with unknown and arbitrary phase shifts^7^.

In above these methods, such as the least-square algorithm (LSA)^8^,^9^, three-step phase-shifting algorithm (3-PSA), four-step phase-shifting algorithm (4-PSA)^10^, five-step phase-shifting algorithm (5-PSA)^11^,^12^, N-step phase-shifting algorithm (N-PSA)^10^, if the phase shifts is known or uniformly distributes in the integer period, these phase retrieval algorithms can work well, otherwise, a large error or deviation of phase retrieval will appear. Typically, if the phase shifts is unknown, there are two phase retrieval solutions: One is to extract the phase shifts in advance, and then retrieve the measured phase with the LSA; the other is to directly retrieve the measured phase without phase shifts extraction. In the former, the main phase shifts extraction algorithm are: arccosine algorithms (ACA)^20^,^21^, 2-norm algorithm^22^, 1-norm algorithm^23^ and inner product algorithm^24^, and then the measured phase can be calculated through using the extracted phase shifts. However, these phase shifts extraction based phase retrieval algorithms not only need a large number of phase-shifting interferograms, but also the computation is time-consuming. Aiming at this situation, some direct phase retrieval algorithms are proposed, such as advanced iterative algorithm (AIA) based on the least-square error estimation and spatial alternative iteration^17^. principal component analysis (PCA)^18^ and independent component analysis (ICA)^19^, etc. Though these direct phase retrieval algorithms reveals high accuracy, but some residual noise and detuning-error still exist, moreover, they are very time-consuming relative to those phase shifts extraction based phase retrieval algorithms.

On the other hand, two orthonormalization two-step phase-shifting algorithm (2-PSA) are developed to improve the speed and accuracy of phase retrieval: One is based on the Gram–Schmidt orthonormalization named as GS^25^, the other is based on the orthogonality of diamond diagonal vectors (DDV)^26^. Though these 2-PSA can work well in the case that the phase shifts is unknown, meanwhile reveals fast computation speed and good consistency while the phase shifts is changed in a large range, but they also need to eliminate the background through filtering processing in advance, otherwise, the error of phase retrieval is large. After that, 2-PSA based on the orthonormalization processing is introduced to three–frame interferograms, the accuracy of phase retrieval can be further improved^27^.

In this study, by using the whole interferogram or local interferogram for computation, a normalization and orthogonalization phase-shifting algorithm (NOPSA or LNOPSA) is proposed to achieve phase retrieval, in which three or more phase-shifting interferograms with unknown phase shifts are employed, the background of interferogram is eliminated through using the orthogonality of complex sinusoidal function in advance, and then the accurate phase can be achieved rapidly through both the normalization and orthogonalization processing. All computations are performed with the CPU of Intel(R) Core(TM) 2 Duo and the 3 GB memory, and with the software package of MATLAB.

## Methods

In PSI, the intensity distribution of the *n*th-frame phase-shifting interferograms can be described as


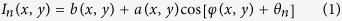


where *b*(*x, y*) and *a*(*x, y*) represent the background intensity and modulation amplitude, respectively; *is φ*(*x, y*) denotes the measured phase; 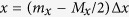 and 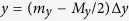 represent the pixel coordinates, in which the center of interferogram is thought as the original point; Δ*x* and Δ*y* are the pixel interval along *x* and *y* direction, respectively; 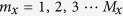 and 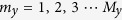 denote the pixel order along the *x* and *y* direction, respectively; *θ*_*n*_ represents the phase shifts and *θ*_0_ = 0, in which 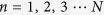 denotes the sequence number of phase-shifting interferograms.

Next, we extract the complex interference term (CIT) of interferograms as following: First, each interferogram is multiplied by 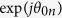. in which 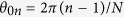 denotes the nominal phase shifts of interferogram; and then the summation operation is performed for all above processed interferograms. Thus, the real part and image part of CIT can be respectively expressed as


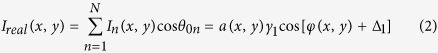


and


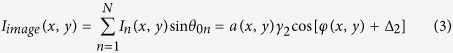


where


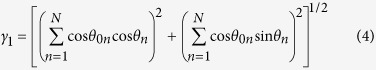



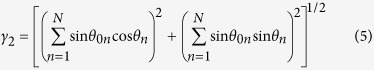



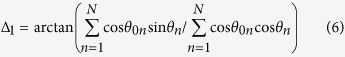



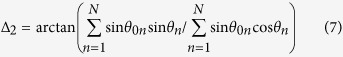


Here, *γ*_1_, *γ*_2_, Δ_1_ and Δ_2_ denote four unknown constants only related with the phase shifts, and 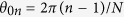
represents the nominal phase shifts. According to the orthogonality of sinusoidal function, in equations (2) and (3), the background of interferogram has been eliminated.

When the actual phase shifts is the same as the nominal phase shifts, we have that 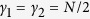, Δ_1_ = 0 and Δ_2_ = *π*/2. Thus, equations (2) and (3) respectively denote the accurate real part and image part of CIT, so the accurate phase can be achieved by using N-PSA^10^. However, if the PSD between the actual phase shifts and nominal phase shifts exists, corresponding to *γ*_1_ ≠ *γ*_2_ and Δ_1_ ≠ Δ_2_, reflecting that the error exists in the real part and image part of CIT, thus the error of phase retrieval will appear. To address this, we perform both the normalization and orthogonalization processing for equations (2) and (3), respectively. For simplicity, we will omit the coordinates (*x, y*) in the following derivation.

Firstly, to avoid the influence of the inconsistency between *γ*_1_ and *γ*_2_, we respectively divide equations (2) and (3) by their root mean square in region 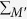 thus


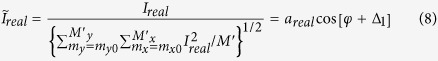


and


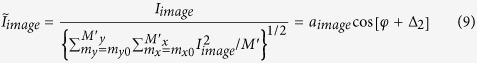


Here, *m*_*x*0_, *m*_*y*0_ denote the initial pixel position in region 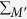, in which 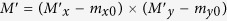. If 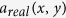 in equation (8) is equal to 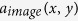 in equation (9), the influence of the inconsistency between *γ*_1_ and *γ*_2_ on the accuracy of phase retrieval can be avoided, thus we have that


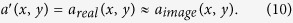


If the fringe number in interferogram is more than one, equation (10) will be well satisfied when the whole interferogram is utilized for computation.

Similarly, in order to avoid the influence of the inconsistency Δ_1_ and Δ_2_, we respectively perform the addition and subtraction operations between equations (8) and (9), thus


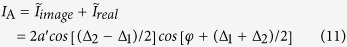


and


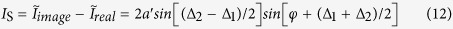


And then we also respectively divide equations (11) and (12) by their root mean square in region 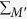, thus


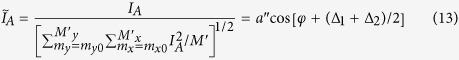



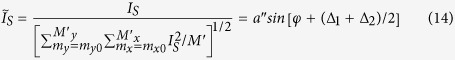


in which


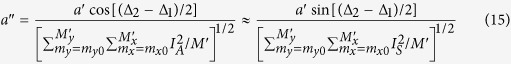


Finally, the measured phase can be calculated by the following expression


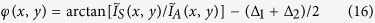


From the above analysis, we can see that even if the PSD exists, the accurate phase also can be achieved through the above processing. That is to say, though the constant (Δ_1_ + Δ_2_)/2 in equation (16) is unknown, but it will not affect the result and accuracy of phase retrieval. For convenience of description, if the whole interferogram is utilized for computation, we name the corresponding processing as the normalization and orthogonalization phase-shifting algorithm (NOPSA); and similarly, the local normalization and orthogonalization phase-shifting algorithm (LNOPSA) is named if a local area of interferogram is utilized for computation.

## Results

Numerical simulation is carried out to verify the effectiveness of the proposed method. Three sequence simulated interferograms with different wavefronts (plane wavefront, complex wavefront and spherical wavefront) are respectively generated. Each sequence includes 4-frame phase-shifting interferograms, and the corresponding phase distributions with and without PSD are calculated by different algorithms. The parameters in equations (4, 5, 6, 7) are respectively set as 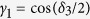, 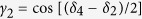, 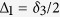 and 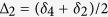, where 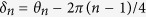 denotes PSD and *δ*_0_ = 0. Moreover, some factors that may influence the performance of the proposed method are analyzed and discussed in this section, such as the fringe number in interferogram and the PSD.

The size of simulated interferograms is set as 512 × 512 pixels, the pixel interval and pixel range are respectively set as 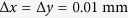 and 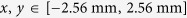; the background and modulation amplitude are 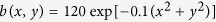 and 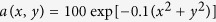, respectively; the nominal phase shifts is equal to *π*/2 and the PSD is set according to the discussion. The phase distributions of three sequence interferograms, are respectively set as the plane wavefront with 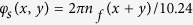, complex wavefront with 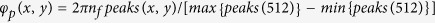 and spherical wavefront with 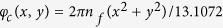, where “peaks” denotes the *peaks* function in Matlab, and 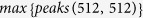 and 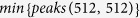 represent the corresponding maximum and minimum of a 512 × 512 matrix. The parameter *n*_*f*_ in phase distribution denotes the fringe number in interferogram. In addition, a Gaussian white noise with mean zero and standard deviation 1 is added to each interferogram. All computations are performed with the CPU of Intel(R) Core(TM) 2 Duo and the 3 GB memory.

**Figure 1:**
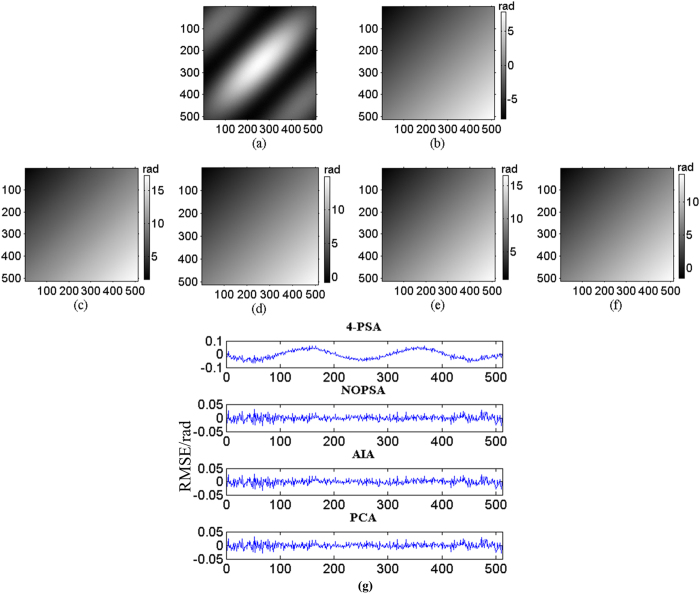
(**a**) One-frame simulated plane wavefront phase-shifting interferogram; (**b**) the theoretical phase; reconstructed phase maps with different algorithms: (**c**) 4-PSA; (**d**) NOPSA; (**e**) AIA; (**f**) PCA; (**g**) the distributions of RMSEs of the differences between the theoretical phase and the retrieved phase (the 256th line) with different algorithms.

**Figure 2:**
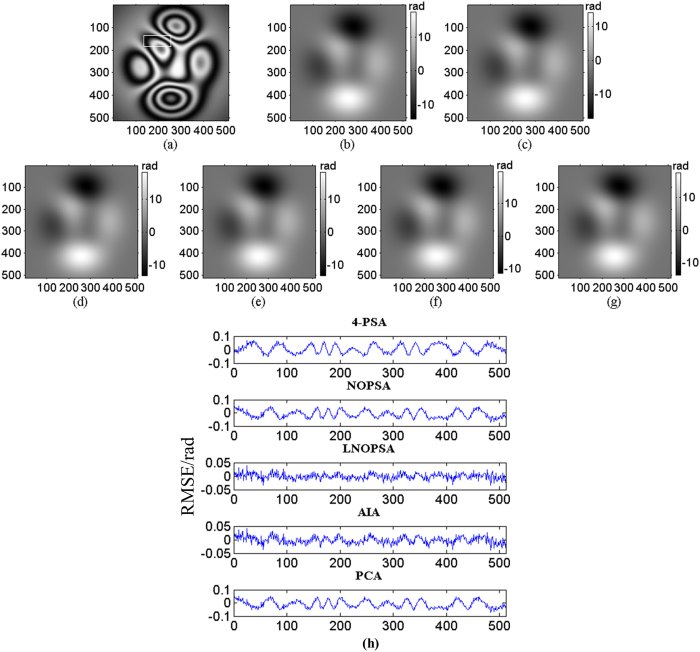
(**a**) One-frame simulated complex wavefront phase-shifting interferogram; (**b**) the theoretical phase; reconstructed phase maps with different algorithms: (**c**) 4-PSA; (**d**) NOPSA; (**e**) LNOPSA; (**f**) AIA; (**g**) PCA; (**h**) the distributions of RMSEs of the differences between the theoretical phase and the retrieved phase (the 256th line) with different algorithms.

**Figure 3:**
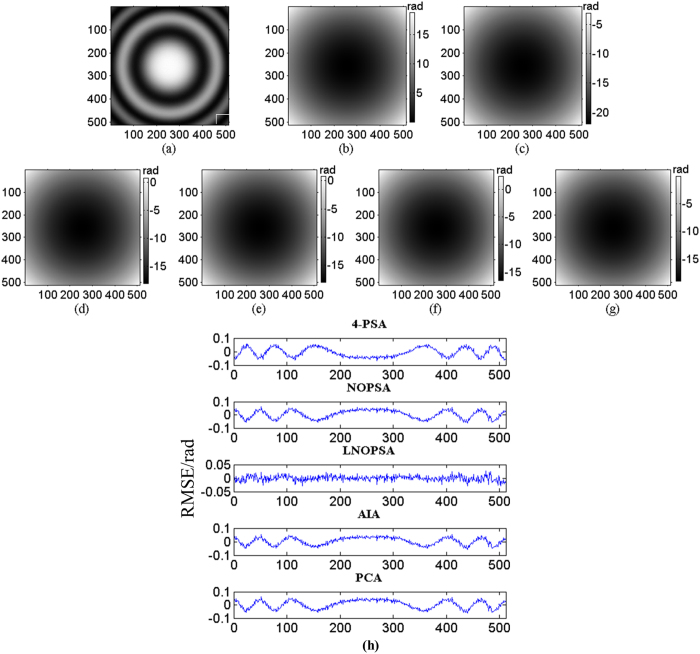
(**a**) One-frame simulated spherical wavefront phase-shifting interferogram; (**b**) the theoretical phase; reconstructed phase maps with different algorithms: (**c**) 4-PSA; (**d**) NOPSA; (**e**) LNOPSA; (**f**) AIA; (**g**) PCA; (**h**) the distributions of RMSEs of the differences between the theoretical phase and the retrieved phase (the 256th line) with different algorithms.

**Figure 4:**
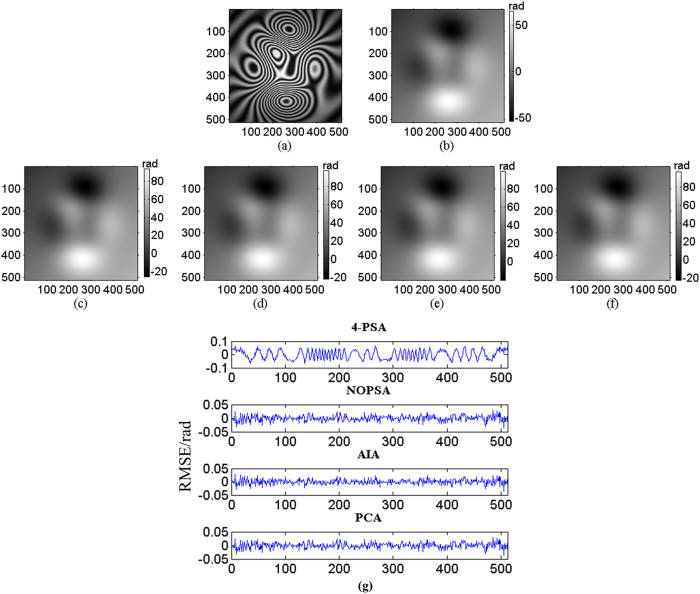
(**a**) One-frame simulated phase-shifting interferogram of complex wavefront with high spatial frequencies; (**b**) the theoretical phase; reconstructed phase maps with different algorithms: (**c**) 4-PSA; (**d**) NOPSA; (**e**) AIA; (**f**) PCA; (**g**) the distributions of RMSEs of the differences between the theoretical phase and the retrieved phase (the 256th line) with different algorithms.

Following, three sequence simulated interferograms with different wavefronts are utilized to perform phase retrieval through the proposed method. The fringe number in interferogram with plane wavefront, complex wavefront and spherical wavefront are set as 2.5, 5 and 3, respectively. The PSD in different interferograms are set as 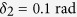, 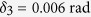 and 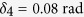. The simulated interferograms and corresponding theoretical phase distributions are shown in Figs 1(a,b), 2(a,b) and 3(a,b). The white squares marked in Figs 2(a) and 3(a) denotes the computation area with LNOPSA algorithm. In complex wavefront, the parameters are set as: 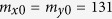, 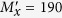, 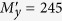. Similarly, the parameters in spherical wavefront are set as: 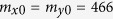 and 
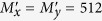
. Meanwhile, the reconstructed phase maps with 4-PSA, NOPSA or LNOPSA, AIA and PCA are shown in Figs 1(c–f), 2(c–g) and 3(c–g), respectively; and the distribution of deviation in the 256th line between the theoretical phase and the retrieved phase with different algorithms are given in Figs 1(g), 2(h) and 3(h), respectively. In addition, we also test the effectiveness of the proposed method through using a more complex phase object with high spatial frequency. The simulated interferogram and corresponding theoretical phase distribution are shown in Fig. 4(a) and (b). The reconstructed phase maps and the distribution of deviation in the 256th line between the theoretical phase and the retrieved phase with different algorithms are given in Fig. 4(c–f) and (g), respectively. Moreover, to compare the errors of the retrieved phase and the computation speed with different algorithms with PSD or without PSD, the root mean square errors (RMSEs) of the difference between the theoretical phase and the retrieved phase, peak to valley error (PVE), as well as the computation time with the NOPSA, LNOPSA, 4-PSA, AIA and PCA algorithms are respectively shown in Table 1, in which the RMSEs are calculated from the whole interferogram.

From the above results, it is found that the proposed algorithm reveals the advantages of rapid speed, high accuracy and good stability, moreover, though the computation time of phase retrieval with the proposed algorithm is almost the same with the 4-PSA algorithm, but it is much less than the AIA and PCA algorithms. Further, even if the PSD exists, the accuracy of phase retrieval with the proposed algorithm is nearly same with the AIA or PCA algorithm but much higher than the 4-PSA algorithm. What is more, we can see that the accuracy of phase retrieval with the NOPSA algorithm is very stable regardless of the PSD is large or small. Specially, by using the plane wavefront interferogram, complex wavefront interferogram with high spatial frequency or the LNOPSA algorithm, the influence of PSD on the accuracy can be eliminated effectively.

Actually, according the derivation of principle in the section “Methods”, we can see that the NOPSA cannot always guarantee that 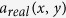 in equation (8) is equal to 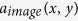 in equation (9), but the LNOPSA can do well, thus the influence of PSD on the accuracy of phase retrieval can be almost eliminated completely through using the LNOPSA. Moreover, in the PCA, if the normalization is not performed, the corresponding accuracy cannot be guaranteed; and in the AIA, if the background in interferograms is not uniform, the accuracy will be affected by PSD. That is to say, due to the accuracy of phase retrieval achieved with the PCA, AIA and NOPSA are related to the fringe number in interferogram, so they are lower than the LNOPSA. In addition, in the computation speed, both the NOPSA and LNOPSA are much faster than the AIA and PCA.

Experimental research is employed to verify the flexibility of the proposed method, in which two wavefront (plane and spherical) interferograms are chosen for phase retrieval by using the proposed NOPSA or LNOPSA, respectively. Mach-Zehnder interferometer based PSI system is constructed to capture phase-shifting interferograms with unknown phase shifts, in which a piezoelectric transducer (PZT) is utilized as the phase-shifting inducer. In one wavelength phase-shifting trip, 136-frame phase-shifting interferograms are captured; and 4-frame and 15-frame phase-shifting interferograms with large and small PSDs are chosen for computation of phase retrieval, respectively. The size of plane wavefront interferogram and spherical wavefront interferogram are equal to 256 × 256 pixels and 350 × 350 pixels, respectively; and the pixel interval of CCD camera is 10 μm × 10 μm. The reference phase (REF) and phase shifts are achieved by using AIA algorithm from 136-frame phase-shifting interferograms.

**Figure 5:**
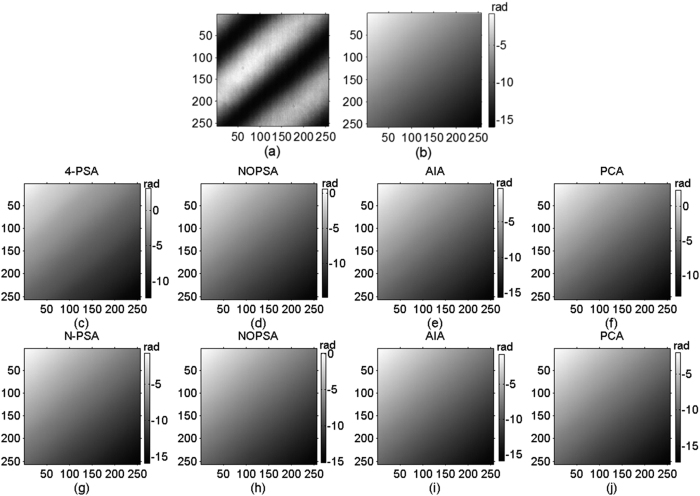
(**a**) One-frame experimental plane wavefront phase-shifting interferogram; (**b**) reference phase (REF); reconstructed phase maps from different number plane wavefront phase-shifting interferograms with large PSDs (**c**–**f**) 4-frame; (**g**–**j**) 15-frame.

**Figure 6:**
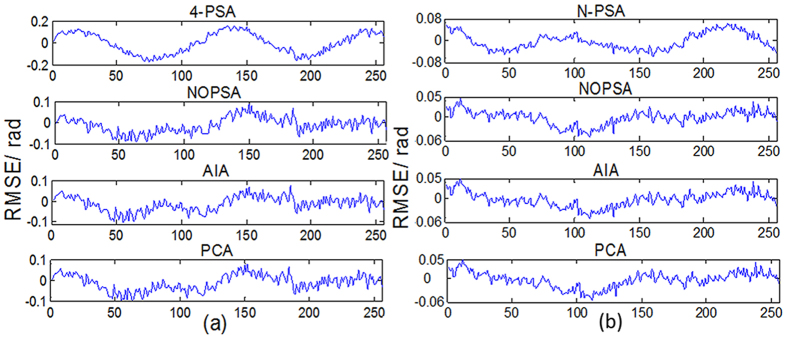
The distributions of RMSEs of the differences between the REF and the phases (the 175th line) respectively retrieved from different number plane wavefront phase-shifting interferograms with large PSDs (**a**) 4-frame; (**b**) 15-frame.

Figure 5(a) and (b) show one-frame experimental plane wavefront interferogram and the corresponding REF, respectively. The reconstructed phase maps from 4-frame and 15-frame plane wavefront phase-shifting interferograms with large PSDs are shown in Fig. 5(c–j). Using the proposed NOPSA, we give the distributions of RMSEs of the differences between the REF and the phases (the 175th line) respectively retrieved from 4-frame and 15-frame plane wavefront phase-shifting interferograms with large PSDs, as shown in Fig. 6(a) and (b). For comparison, Fig. 6 also show the corresponding results achieved with the N-PSA, AIA and PCA, respectively.

**Figure 7:**
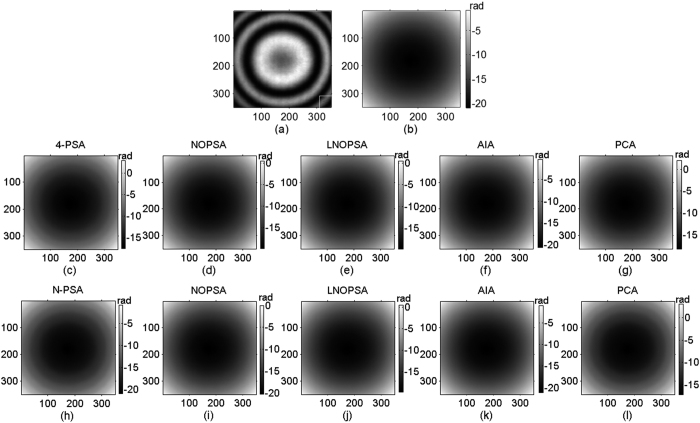
(**a**) One-frame experimental spherical wavefront phase-shifting interferogram; (**b**) reference phase (REF); reconstructed phase maps from different number spherical wavefront phase-shifting interferograms with large PSDs (**c**–**g**) 4-frame; (**h**–**l**) 15-frame.

**Figure 8:**
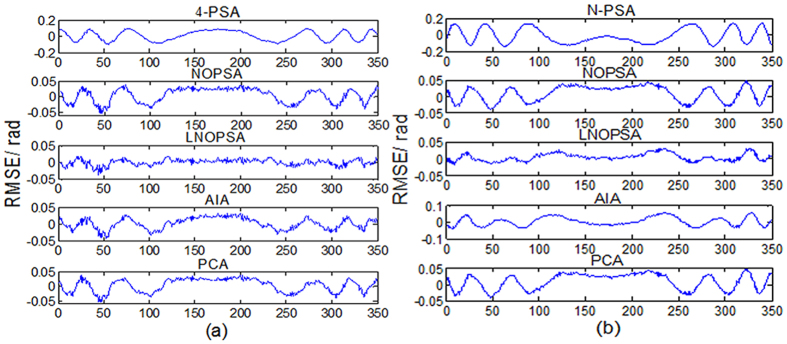
The distributions of RMSEs of the differences between the REF and the phases (the 175th line) respectively retrieved from different number spherical wavefront phase-shifting interferograms with large PSDs (**a**) 4- frame; (**b**) 15- frame.

Figure 7(a) and (b) respectively give one-frame experimental spherical wavefront phase-shifting interferogram and the corresponding REF, in which the white square marked in Fig. 7(a) denotes the computation area utilized by LNOPSA. The reconstructed phase maps from 4-frame and 15-frame spherical wavefront phase-shifting interferograms with large PSDs are given in Fig. 7(c–l). Similarly, the distributions of RMSEs of the differences between the REF and the phases (the 175th line) are shown in Fig. 8.

For 4-frame phase-shifting interferograms, the nominal phase shifts are set as *θ*_01_ = 0, *θ*_02_ = π/2, *θ*_03_ = π and *θ*_04_ = 3π/2, respectively. In the plane wavefront interferograms, the large and small PSDs are 0.83 rad, 0.4029 rad, 0.0012 rad, and 0.0071 rad, 0.017 rad, 0.01 rad, respectively; and in the spherical wavefront interferograms, the large and small PSDs are 0.587 rad, −0.010 rad, −0.4211 rad, and 0.0021 rad, −0.01 rad, −0.07 rad, respectively.

For 15-frame phase-shifting interferograms, we set the nominal phase shifts as 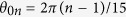. In the plane wavefront interferograms, the RMSEs with large and small PSDs are 0.1948 rad and 0.020 rad, respectively; and in the spherical wavefront interferograms, the RMSEs with large and small PSDs are 0.3881 rad and 0.0287 rad, respectively.

Subsequently, we present an overall comparison about the error and computation speed of phase retrieval with different algorithms. In both large and small PSDs, from 4-frame phase-shifting interferograms, Table 2 gives the RMSE of the difference between the REF and the retrieved phase, PVE, as well as the computation time with the NOPSA, LNOPSA, 4-PSA, AIA and PCA algorithms, respectively. Similarly, the results achieved from 15-frame phase-shifting interferograms are shown in Table 3.

As be seen in Figs 6 and 8, Tables 2 and 3, the performance of NOPSA is very similar with AIA and PCA, indicating that using the NOPSA, the influence of PSD on accuracy of phase retrieval can been almost eliminated completely. In the case that the PSD is large, the RMSE of phase retrieval with the NOPSA is much less than the N-PSA, revealing the better stability of the NOPSA; and when the PSD is small, the RMSE of phase retrieval with the NOPSA is almost the same with N-PSA algorithm. In particular, it is found that using the proposed LNOPSA, the influences of interferogram quantity, fringe shape and PSD on the accuracy of phase retrieval are almost eliminated completely. In addition, we can see that though the computation time with the NOPSA is slightly more than the traditional N-PSA, but along with the number increasing of interferograms, the difference of computation time between the NOPSA and N-PSA can be ignored. Further, it is also presented that the computation time with the NOPSA is much less than AIA and PCA regardless of the number of interferograms.

## Discussions

**Figure 9:**
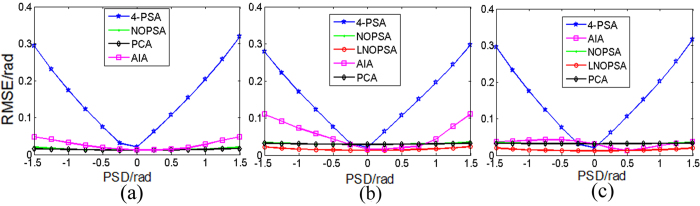
Relationship between the RMSE of phase retrieval with different algorithms and the PSD for different wavefront interferograms (**a**) plane wavefront; (**b**) complex wavefront; (**c**) spherical wavefront.

In order to further present the performance of the proposed NOPSA algorithm in different PSDs, in this section, these interferograms as utilized in Figs (1, 2 and 3) are generated again, in which the phase shifts of the 2st and 3th-frame interferograms are respectively set as 0.1 and 0.006 rad while the phase shifts of the 4th- frame interferograms is changed from −1.5 to 1.5 rad. Figure 9 gives the relationship between the RMSE of phase retrieval with NOPSA, LNOPSA, 4-PSA, AIA and PCA algorithms and the phase shifts of the 4th frame interferogram. Obviously, we can see that the RMSE of phase retrieval with 4-PSA algorithm is increased with the PSD; and though the influence of PSD on accuracy of phase retrieval can be eliminated effectively with AIA or PCA algorithm, the RMSE of phase retrieval with AIA algorithm is increased with the PSD, so the performance becomes unstable. In contrast, the good consistency and high accuracy with NOPSA algorithm can be achieved while the PSD is changed from 0 to 1.5 rad. In particular, we can see that the influence of PSD on accuracy is almost eliminated by using the proposed LNOPSA algorithm. These results further demonstrate the obvious advantages of the proposed algorithm in avoiding the influence of PSD, high accuracy and good stability.

**Figure 10:**
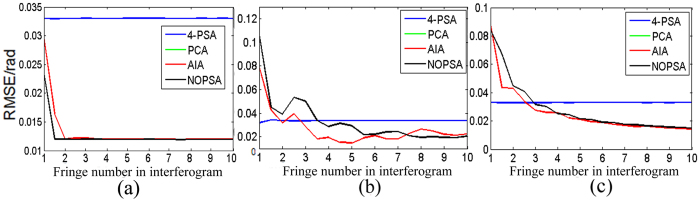
Relationship between the RMSE of phase retrieval with different algorithms and the fringe number in interferogram with different wavefronts (**a**) plane wavefront; (**b**) complex wavefront; (**c**) spherical wavefront.

Note that though the proposed algorithm reveals strong ability to eliminate the influence of PSD, but its accuracy depends on the fringe number *n*_*f*_ in interferogram. To address this, we change the fringe number in interferogram, and then perform the phase retrieval of the above interferograms through using the proposed algorithm. Assuming that 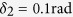, 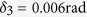 and 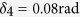; and *n*_*f*_ is changed from 1 to 10, a Gaussian white noise with mean zero and standard deviation 1 is added to each interferogram. By using the 4-PSA, AIA, PCA and NOPSA to perform phase retrieval, we achieve the corresponding results, as shown in Fig. 10. It is found that for the plane wavefront phase-shifting interferograms, the RMSE of phase retrieval with the NOPSA is almost unchanged (0.012 rad) when the fringe number is more than 1.2; and for the complex wavefront and spherical wavefront phase-shifting interferograms, the RMSE of phase retrieval with the NOPSA is less than 0.1 rad for *n*_*f*_ > 2 and less than 0.02 rad for *n*_*f*_ > 5. In addition, it is also presented that the accuracy of phase retrieval with AIA or PCA depends on the fringe number in interferogram, and the corresponding variation curve is very similar with the NOPSA algorithm. From these results, we can conclude that the proposed NOPSA method will provide a powerful solution to eliminate the influence of PSD on the accuracy of phase retrieval if the fringe number in interferogram is enough.

## Conclusion

In summary, based on both normalization and orthogonalization processing, we propose an advanced NOPSA (or LNOPSA) to perform phase retrieval in PSI with unknown phase shifts, in which the background of interferogram is eliminated by using the orthogonality of complex sinusoidal function, and then the influence of PSD on accuracy of phase retrieval is avoided through both the normalization and orthogonalization processing. The main advantages of the proposed algorithm are as following: First, it is not needed to perform the least square estimation and phase shifts extraction. Second, the influences of interferogram quantity, fringe shape and PSD on the accuracy of phase retrieval can be almost eliminated completely through using the proposed LNOPSA. Further, compared with the current algorithms, the proposed algorithm reveals significantly faster speed, higher accuracy, better stability and non-sensitivity of PSD while its application condition is equivalent to AIA and PCA algorithms. To the best of our knowledge, in addition to maintaining the advantage of high accuracy, the proposed algorithm show the fastest speed in current PSAs with unknown phase shifts, and the LNOPSA is an almost perfect solution in eliminating the influence of PSD on the accuracy while its calculation area determination still needs to be investigated further.

## Additional Information

**How to cite this article**: Li, J. *et al*. An advanced phase retrieval algorithm in N-step phase-shifting interferometry with unknown phase shifts. *Sci. Rep.* **7**, 44307; doi: 10.1038/srep44307 (2017).

**Publisher's note:** Springer Nature remains neutral with regard to jurisdictional claims in published maps and institutional affiliations.

## Figures and Tables

**Table 1:** RMSE (rad), PVE (rad) and Time (s) of phase retrieval with different algorithms (Simulation).

		NOPSA	LNOPSA	4-PSA	AIA	PCA	NOPSA	LNOPSA	4-PSA	AIA	PCA
		(with PSD)					(without PSD)				
**Plane Wavefront**	**RMSE**	0.012	/	0.033	0.013	0.012	0.012	/	0.012	0.012	0.012
	**PV**	0.163	/	0.231	0.166	0.163	0.180	/	0.181	0.179	0.180
	**Time**	0.057	/	0.033	110.7	0.169	0.057	/	0.032	12.32	0.162
**Complex Wavefront**	**RMSE**	0.029	0.013	0.034	0.015	0.029	0.029	0.013	0.012	0.017	0.029
	**PV**	0.199	0.158	0.208	0.160	0.199	0.204	0.164	0.162	0.167	0.204
	**Time**	0.058	/	0.033	112.4	0.164	0.058	/	0.033	110.5	0.169
**Spherical Wavefront**	**RMSE**	0.032	0.012	0.033	0.028	0.032	0.032	0.013	0.012	0.026	0.032
	**PV**	0.220	0.164	0.226	0.204	0.220	0.224	0.188	0.183	0.198	0.224
	**Time**	0.056	/	0.033	110.2	0.165	0.059	/	0.034	110.3	0.161
**High Frequency Object**	**RMSE**	0.013	/	0.033	0.012	0.013	0.013	/	0.012	0.012	0.013
	**PV**	0.153	/	0.223	0.147	0.153	0.163	/	0.162	0.162	0.163
	**Time**	0.056	/	0.034	111.6	0.167	0.060	/	0.031	109.5	0.162

**Table 2:** RMSE (rad), PVE (rad) and Time (s) of phase retrieval with different algorithms from 4-frame interferograms (Experiment).

		NOPSA	LNOPSA	4-PSA	AIA	PCA	NOPSA	LNOPSA	4-PSA	AIA	PCA
		(with large PSD)					(with small PSD)				
**Plane Wavefront**	**RMSE**	0.037	/	0.087	0.038	0.038	0.029	/	0.029	0.029	0.029
	**PV**	0.282	/	0.405	0.286	0.253	0.218	/	0.210	0.210	0.215
	**Time**	0.013	/	0.008	27.55	0.034	0.012	/	0.010	4.535	0.035
**Spherical Wavefront**	**RMSE**	0.023	0.012	0.060	0.018	0.023	0.023	0.013	0.023	0.027	0.023
	**PV**	0.147	0.111	0.274	0.138	0.145	0.158	0.126	0.150	0.181	0.160
	**Time**	0.024	/	0.015	26.57	0.071	0.024	/	0.016	55.38	0.074

**Table 3:** RMSE (rad), PVE (rad) and Time (s) of phase retrieval with different algorithms from 15-frame interferograms (Experiment).

		NOPSA	LNOPSA	N-PSA	AIA	PCA	NOPSA	LNOPSA	N-PSA	AIA	PCA
		(with large PSD)					(with small PSD)				
**Plane Wavefront**	**RMSE**	0.017	/	0.032	0.018	0.018	0.020	/	0.021	0.019	0.020
	**PV**	0.127	/	0.171	0.130	0.136	0.138	/	0.140	0.137	0.137
	**Time**	0.033	/	0.028	8.737	0.296	0.034	/	0.030	6.939	0.297
**Spherical Wavefront**	**RMSE**	0.022	0.010	0.097	0.023	0.022	0.022	0.010	0.015	0.011	0.022
	**PV**	0.125	0.091	0.329	0.117	0.125	0.118	0.086	0.101	0.111	0.116
	**Time**	0.071	/	0.062	63.36	0.560	0.070	/	0.062	60.39	0.560
